# Effective Young’s Modulus Estimation of Natural Fibers through Micromechanical Models: The Case of Henequen Fibers Reinforced-PP Composites

**DOI:** 10.3390/polym13223947

**Published:** 2021-11-15

**Authors:** Ferran Serra-Parareda, Fabiola Vilaseca, Roberto Aguado, Francesc X. Espinach, Quim Tarrés, Marc Delgado-Aguilar

**Affiliations:** 1 LEPAMAP-PRODIS Research Group, University of Girona, C/Maria Aurèlia Capmany 61, 17003 Girona, Spain; ferran.serrap@udg.edu (F.S.-P.); roberto.aguado@udg.edu (R.A.); francisco.espinach@udg.edu (F.X.E.); 2 Advanced Biomaterials and Nanotechnology, Department of Chemical Engineering, University of Girona, Maria Aurèlia Capmany 61, 17003 Girona, Spain; fabiola.vilaseca@udg.edu

**Keywords:** composites, Young’s modulus, stiffness, henequen, polypropylene

## Abstract

In this study, Young’s modulus of henequen fibers was estimated through micromechanical modeling of polypropylene (PP)-based composites, and further corroborated through a single filament tensile test after applying a correction method. PP and henequen strands, chopped to 1 mm length, were mixed in the presence of maleic anhydride grafted polypropylene (MAPP). A 4 wt.% of MAPP showed an effective enhancement of the interfacial adhesion. The composites were mold-injected into dog-bone specimens and tensile tested. The Young’s modulus of the composites increased steadily and linearly up to 50 wt.% of fiber content from 1.5 to 6.4 GPa, corresponding to a 327% increase. Certainly, henequen fibers showed a comparable stiffening capacity of PP composites than glass fibers. The intrinsic Young’s modulus of the fibers was predicted through well established models such as Hirsch or Tsai-Pagano, yielding average values of 30.5 and 34.6 GPa, respectively. The single filament test performed to henequen strands resulted in values between 16 and 27 GPa depending on the gauge length, although, after applying a correction method, a Young’s modulus of 33.3 GPa was obtained. Overall, the present work presents the great potential for henequen fibers as PP reinforcement. Moreover, relationships between micromechanics models and filament testing to estimate Young’s modulus of the fibers were explored.

## 1. Introduction

In recent years, natural fibers have experienced growing demand as plastic reinforcement/filler as a result of the growing environmental consciousness in our society and the need amongst manufacturers for eco-friendly materials [1,2]. Natural fibers are viewed as a sustainable alternative to conventional synthetic fibers (i.e., glass, carbon, aramid) because of their biobased, biodegradable, renewable, and recyclable nature. Further, natural fibers have a low density (around 1.35–1.55 g/cm^3^), can be purchased at relatively low cost, are non-abrasive for the processing equipment, and are non-harmful to human beings [3,4,5,6]. The incorporation of natural fibers in thermoplastic matrices has shown a reasonable enhancement of the mechanical properties in terms of stiffness and rigidity. However, the strength improvement is more reliant on the fiber-matrix interfacial characteristics [7]. These characteristics shown by natural fiber polymer composites have made them suitable for use in various fields such as automotive and building and construction, their use being especially attractive for applications demanding low-density materials [8,9]. In general, it is suggested that the sustainable character of natural fibers may contribute to ecosystem health and sustainability, whereas their low cost, ease of processability, and physicomechanical performance may fulfill the technical and economic interest of industry [10]. Natural fibers can be obtained from different lignocellulosic feedstocks such as wood [11,12], annual plants [13,14,15], agroforestry residues [16,17], or recycled paper and board products [18,19]. In this work, the case of henequen (*Agave fourcroyedes*), which is native from Yucatan (Mexico) and a close relative to the sisal plant (*Agave sisalana*), was studied and evaluated as polypropylene (PP) reinforcement.

Despite natural fibers offering many benefits, the lack of compatibility between the lignocellulosic and polymeric materials poses a barrier to the full development of the composites’ properties [20]. Such incompatibility is explained by the different surface chemistry of natural fibers and thermoplastic materials. For instance, polyolefins such as polypropylene (PP) and polyethylene (PE) are non-polar and hydrophobic. Moreover, natural fibers have polar groups (i.e., hydroxyl groups) at their surface that provide hydrophilicity to these materials. Consequently, the fibers may self-aggregate via hydrogen bonding and be unevenly dispersed throughout the matrix phase, particularly at elevated fiber contents. The stress-transfer capacity may also drop considerably given the lack of fiber-matrix adhesion, finally affecting the mechanical properties of the material [21]. The scarce fiber-matrix compatibility may also lead to insufficient wetting of the fibers, contributing to voids formation, and decreasing the water and gas barrier properties [22]. Overall, addressing the issue of fiber-matrix compatibility and poor interfacial adhesion has been one of the most challenging tasks in the composite sector [23]. 

The above-mentioned shortcomings of natural fiber composites can be avoided, or at least mitigated, by surface modification of the fibers (i.e., alkaline, acetylation, silane, graft copolymerization treatments, among others) to make them more hydrophobic [24], or by the addition of coupling agents that promote the interactions between both phases [25]. The latter seems to be a more cost-effective and efficient manner, at least on the basis of large-scale production, to address the issue of the adhesion between fibers and matrix. In the case of polypropylene (PP)-based composites, the use of maleic anhydride grafted polypropylene (MAPP) has shown effective enhancement of the fiber-matrix compatibility [26,27,28]. Briefly, MAPP’s interaction mechanism is based on the formation of chemical bonds via esterification with the hydroxyl groups at the fiber surface. In addition, the PP chains from MAPP may diffuse through the PP matrix via a self-entangling mechanism (physical interactions) [29]. Soleimani et al. [30] stated that the addition of a 5 wt.% of MAPP to flax fiber-reinforced PP composites improved the physical and mechanical properties (water absorption, tensile strength, and impact strength). Nayak et al. reported that the presence of MAPP combined with NaOH treated sisal fibers incremented the tensile strength by 28.22 % in comparison to the uncoupled composite, with almost negligible effects on the tensile modulus [31]. 

As explained, improving the fiber-matrix compatibility is required to obtain technically competitive materials. It is accepted that for engineering and structural applications, the most relevant properties to consider with natural fiber composites are stiffness and dimensional stability [32,33]. The stiffness of materials is generally evaluated by Young’s modulus parameter, which is in essence the slope of the line at the elastic zone of the tensile stress-strain curve. The Young’s modulus of the composite can be modeled through micromechanics analysis to study the relationships within composite microstructure in terms of, for example, morphology, orientation, and fibers’ intrinsic properties [34,35]. Micromechanical modeling also allows the prediction of the anisotropic behavior of the composites, which is deemed useful to evaluate the performance of the materials under different loading conditions [36]. A more straightforward manner of determining the intrinsic mechanical properties of the fibers is by submitting them, at the indicated conditions and specifications, to a single filament tensile test. However, the process is tedious since many tests must be performed to attain reliable results, due to the irregular shape and morphology of the strands, which generally leads to high standard deviations. Further, the testing equipment may induce an error in the measurement of Young’s modulus. As a consequence, the literature shows that the intrinsic Young’s modulus obtained from raw fibers via single fiber testing, and from the fibers inside the composite through mathematical modeling, can be noticeably different [37]. 

In brief, in this study, composite materials based on PP, henequen strands chopped at 1 mm length, and MAPP were prepared at fiber contents ranging from 10 to 50 wt.%. The stiffness of the composites, reflected in Young’s modulus parameter, were evaluated both at macro and micro scales. In parallel, henequen strands were submitted to a single filament test to evaluate its Young’s modulus, the values of which have been posteriorly corrected as for the error induced by the equipment-specimen interactions. In general, the present works stand as a thoughtful and innovative study on the potential of henequen strands as PP reinforcement in substitution to conventional synthetic fibers and on the relationships of the intrinsic Young’s modulus of natural fibers obtained via micromechanics models and direct tensile testing.

## 2. Materials and Methods

### 2.1. Materials

The polymer matrix was a PP (ISPLEN PP090 G2M) provided by (Repsol, Tarragona, Spain). The injection grade PP presents a melt flow index of 30 g/10 min (230 °C; 2.16 kg) and a density of 0.905 g/cm^3^. A modified MAPP (Epolene G3015) was incorporated into the materials as a coupling agent. MAPP was acquired from (Eastman Chemical Products, San Roque, Spain).

Henequen strands from Agave (*Agave fourcroyedes*) were kindly supplied by Centro de Investigación Científica de Yucatán (CICY) (Mérida, Mexico). The lignocellulosic material contains 68.1% of cellulose, 18.2% of hemicellulose, 8.7% of lignin, 3.7% of extractives, and 1.3% of ashes. The strands were initially chopped to 1 mm in length without any further treatments. The average diameter of the strands was previously determined by optical microscopy at 220.8 μm [38]. The obtained fibrous-like material was effectively blended with PP at different percentages.

### 2.2. Methods

**Figure 1:**
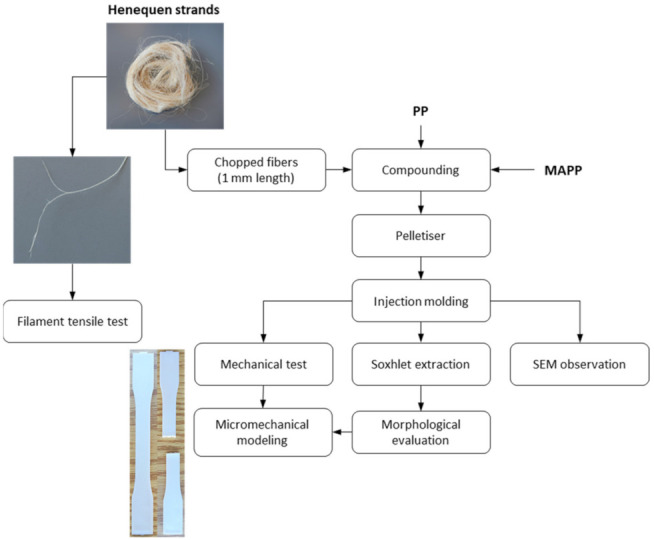
Workflow of the present investigation.

The work plan of the present investigation is briefly schematized in Figure 1.

#### 2.2.1. Preparation of Composite Materials

Composite materials were prepared using a Brabender Plastograph mixer (Brabender^®^, Duisburg, Germany) equipped with two counter-rotating screws to obtain a well-dispersed material. First, PP and MAPP were introduced together to the mixing chamber at 180 °C and 80 rpm until the mixture was completely melted. Henequen fibers, chopped at 3 mm length, were then incorporated into the process by keeping constant the temperature and rotation speed. Reduction of henequen strands length is crucial to avoid entanglement between the strands. The reduction of the length of the henequen filament allows a correct dispersion of the fibers and therefore homogeneity of the composite material. The resulting mixture was discharged from the mixing chamber, cooled down, and grounded using a knife mill equipped with a 5 mm mesh screen at the bottom to ensure the size homogeneity of the granules. The obtained granules were stored at 80 °C for 24 h to remove the remaining moisture.

#### 2.2.2. Injection Molding of Standard Specimens and Mechanical Characterization

Dog-bone specimens were produced with a steel mold in an injection molding machine Meteor-40 (Mateu and Solé, Spain). The injection process was carried out at temperatures of 175 °C at zone 1, 175 °C at zone 2, and 190 °C in the nozzle, regarding the three heating areas of the injection machine, in addition to what is required by ASTM D3641. Before tensile testing, the dog-bone specimens were kept in a climate control chamber at 23 °C and 50% of relative humidity (during 48 h), according to ASTM D618. The tensile properties were determined using a universal testing machine (INSTRON 1122) working at 2 mm/min, according to ASTM D638 specifications. The Young’s modulus was evaluated using an MFA 2 extensometer (Velbert, Germany) in dog-bone specimens for a more precise elongation measurement.

#### 2.2.3. Morphological Analysis of the Fibers

During the compounding processes, fibers may suffer some morphological changes. As a result, it is important to evaluate the morphology of the fibers after compounding. To this end, the composite materials were submitted to a Soxhlet extraction, using decahydronaphthalene (decalin) as the solvent, to dissolve PP and recover the reinforcing fibers. The morphological features of the fibers, mainly length and diameter, were determined using a morphological analyzer MorFi Compact from Techpap SAS (Grenoble, France).

#### 2.2.4. Scanning Electron Microscopy

Henequen strands were observed by scanning electron microscopy (SEM) (Zeiss DMS 960) to allow a qualitative evaluation of the appearance and irregularities in the shape of the strand.

#### 2.2.5. Single Filament Test

**Figure 2:**
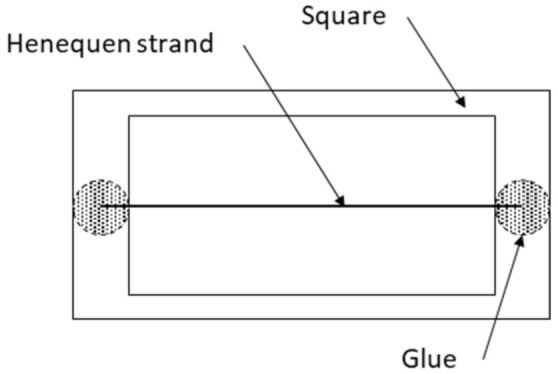
Filaments characterizing method.

The characterization of the filaments consisted of selecting filaments at random and adhering them individually on cardboards with rectangular orifices of equal measurement, as is shown in Figure 2. The filaments were carefully aligned and held with glue. According to the specific normative of the test described in standard ASTM D3822, the test was performed at different gauge lengths of 1, ¾, ½, and ¼ inches (25.4, 19.05, 12.7, and 6.35 mm, respectively).

## 3. Results and Discussion

### 3.1. Coupling Agent Optimization

**Figure 3:**
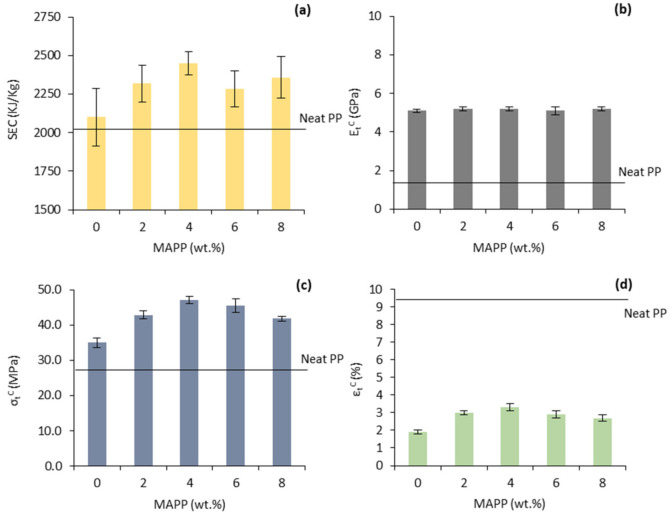
Effect of MAPP addition on the 40 wt.% henequen fibers-reinforced PP composites: (**a**) Specific energy consumption; (**b**) Young’s modulus (E_t_^C^); (**c**) tensile strength (σ_t_^C^); (**d**) elongation at maximum stress (ε_t_^C^).

The inherent incompatibility between henequen fibers and polypropylene (PP) may inevitably lead to weak interfacial adhesion, scarce stress-transfer capacity, creation of void spaces, and poor water barrier properties. Such issues can be avoided, or at least mitigated, by incorporating maleic anhydride grafted polypropylene (MAPP) into the composites, which may act as a bridge between the lignocellulosic and polymeric phases. MAPP’s interaction mechanism has been thoroughly illustrated and discussed in previous works [39,40]. Figure 3 shows the effect of different MAPP percentages (from 2 to 8 wt.%) on specific energy consumption (SEC) and the tensile properties (strength, Young’s modulus, and elongation at break) for 40 wt.% henequen fibers-reinforced PP composites.

From Figure 3a one can see that the SEC followed a similar trend to strength and elongation. In general, energy consumption may increase with melt viscosity due to the increased torque. Hence, it is suggested that the viscosity of the composites containing a 4 wt.% of MAPP was higher than in the other materials probably due to the improved adhesion and dispersion of the phases, finally contributing to energy consumption.

Young’s moduli of the 40 wt.% reinforced composites were remarkably higher than that of unreinforced PP (1.5 ± 0.1 GPa) and almost independent of the amount of MAPP. Figure 3b shows that the mean value of Young’s moduli remained almost the same despite the presence or the percentages of MAPP. Then, the presence of MAPP did not substantially affect Young’s modulus of the composites. This agrees with the literature which states that the stiffness of composite materials should not be affected by the quality at the interfacial boundary, and thus, by the presence of coupling agents [39,41]. 

From Figure 3c, it is observed that the tensile strength of the composite without MAPP was close to the neat matrix (27.6 ± 0.5 MPa), evidencing scare fiber-matrix compatibility. The addition of MAPP progressively increased the tensile strength up to a 4 wt.% of the coupling agent, where the tensile strength reached 47.2 ± 1.0 MPa (+ 71% increment). At higher MAPP contents, the strength decreased again, which could indicate the saturation of the hydroxyl groups, resulting in self-entanglement and slippage of MAPP molecules [41,42]. The elongation at maximum stress (Figure 3d) followed a similar tendency to the tensile strength. In this case, the parameter increased from 1.9 % (0 wt.% MAPP) to 3.3 % (4 wt.% MAPP). It is worth noting that MAPP may favor the adequate dispersion and distribution of henequen fibers throughout the matrix phase by avoiding fibers’ self-aggregation, and this could also enhance the strength and elongation at break properties of the material. 

### 3.2. Analysis of the Young’s Modulus

**Figure 4:**
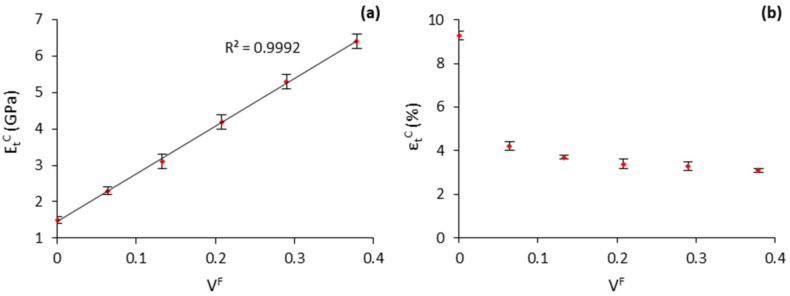
Evolution of the (**a**) Young’s modulus ($E_{t}^{C}$); (**b**) elongation at maximum stress ($\varepsilon_{t}^{C}$) with the fiber volume fraction (V^F^).

PP-based composites with varying amounts of henequen fibers, from 10 to 50 wt.%, and 4 wt.% of MAPP were prepared. Table 1 presents the evolution of Young’s modulus and elongation at maximum stress of the composites. Further, the evolution of these properties is represented with the fiber volume content in Figure 4.

The Young’s modulus of the composites increased steadily and linearly (Figure 4) with the fiber volume fraction from 1.5 GPa (neat PP) to 6.4 GPa (50 wt.% reinforced composite). The linear increase of the mechanical property with the fiber content has been regarded as an indicator of adequate dispersion and distribution of the fibers within the polymeric phase. This is considered a remarkable outcome since some cellulose-rich lignocellulosic fillers/fibers tend to aggregate at elevated percentages, critically decreasing their mechanical performance. Indeed, it has been reported that a 15–25 wt.% of fiber content is optimal for polyester and polyolefin-based composites [43,44,45]. Thereby, the fact that the composites could be effectively charged up with a 50 wt.% of henequen fibers, with no significant drop in their elongation capacity, is considered relevant especially for the obtention of low-cost and mechanically competitive materials. The composites showed comparable stiffness to those glass fiber (GF)-reinforced PP composites. For instance, the 30 wt.% henequen-reinforced composites exhibited similar Young’s modulus than a composite containing a 40 wt.% of GFs, yet offering the advantage of lower-weight, and more environmentally friendly material [46,47,48]. The manufactured composites also showed a greater stiffening potential than other natural fiber-based composites from wood resources [49].

### 3.3. Fiber Contribution to the Young’s Modulus

The stiffening potential of henequen fibers can be trustfully compared with other types of fibers by defining a Fiber Tensile Modulus Factor (FTMF). The FTMF can be obtained by rearranging the well-known modified Rule of Mixtures (mRoM). The mRoM combines the contribution of the reinforcement and matrix, separately, to Young’s modulus of a composite, as expressed in Equation (1).

(1) $$E_{t}^{C}=\eta_{e}\cdot E_{t}^{F}\cdot V^{F}+E_{t}^{M}\cdot (1-V^{F});\;\eta_{e}=\eta_{o}\cdot \eta_{l}$$

In Equation (1), Young’s modulus of the composite, fibers, and matrix are represented by $E_{t}^{C}$, $E_{t}^{F}$, and $E_{t}^{M}$, respectively. $\eta_{e}$ represents the modulus efficiency factor and such factor is introduced to the model to correct the stiffening efficiency of the fibers. Such factor principally depends on the orientation and morphological features of the fibers and hence can be expressed as the product between a modulus orientation factor ($\eta_{o}$) and modulus length factor ($\eta_{l}$). The FTMF can be obtained by isolating the contribution of the fibers to Young’s modulus of the composite, described by $\eta_{e}\cdot E_{t}^{F}$, as shown in Equation (2).

(2) $$\mathrm{FTMF}=\eta_{e}\cdot E_{t}^{F}=\frac{E_{t}^{C}-E_{t}^{M}\cdot \left(1-V^{F}\right)}{V^{F}}$$

The FFMF is obtained from the slope of the line by representing $E_{t}^{C}-E_{t}^{M}\cdot \left(1-V^{F}\right)$ against $V^{F}$ at each fiber content. The FFMF is deemed an adequate indicator of the stiffening potential of the fibers and can be used for comparison purposes. In this work, the FFMF obtained for henequen fibers was compared to other fiber typologies such as wood fibers [49] and glass fibers [46], which were selected as main representatives inside the categories of natural and synthetic fibers.

**Figure 5:**
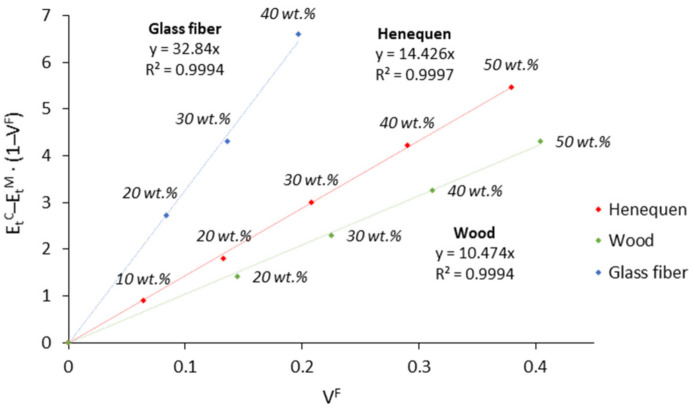
FFMF of henequen fibers, wood fibers, and glass fibers in polypropylene composites.

The FFMF of henequen, glass, and wood fibers were 14.4, 32.8, and 10.5, respectively. Out of these values, it is possible to estimate that by keeping constant the fiber volume fraction, wood fibers and glass fibers should have a stiffening effect of 0.73 and 2.3 in comparison to henequen fibers. Regarding weight percentages, Figure 5 states that 50 wt.% of wood fibers, 40 wt.% of henequen fibers, and 30 wt.% of glass fibers can stiffen PP similarly.

### 3.4. Estimation of the Intrinsic Young’s Modulus of Henequen Fibers

The modulus efficiency factor ($\eta_{e}$) and intrinsic Young’s modulus of the fibers are unknown variables in the mRoM. A possible way to estimate the intrinsic Young’s modulus natural fibers is through micromechanical modeling. Two well-established models that have offered an effective prediction of such intrinsic property are Hirsch and Tsai-Pagano models. Tsai-Pagano model can be combined with Halpin-Tsai equations, hereby abbreviated as TP & HT model. The Hirsch model is described in Equation (3) [50].

(3) $$E_{t}^{C}=\beta \cdot \left(E_{t}^{F}\cdot V^{F}-E_{t}^{M}\left(1-V^{F}\right)\right)+\left(1-\beta \right)\cdot \frac{E_{t}^{F}\cdot E_{t}^{M}}{E_{t}^{M}\cdot V^{F}+E_{t}^{F}\cdot \left(1-V^{F}\right)}$$

The factor β is introduced to the Hirsch model to correct the contribution of the fibers to Young’s modulus. β values close to 0.4 have proved to adequately reproduce the results obtained experimentally for natural fiber composites processed using injection molding [51]. Unlike the Hirsch model, the Tsai-Pagano model in combination with Halpin-Tsai equations incorporates morphological data from the fibers [52]. Tsai-Pagano model is shown in Equation (4) [53], whereas the respective longitudinal modulus ($E^{11}$) and transverse modulus ($E^{22}$) are computed according to Halpin-Tsai equations, as shown in Equations (5) and (6), respectively [54].

(4) $$E_{t}^{C}=\frac{3}{8}\cdot E^{11}+\frac{5}{8}\cdot E^{22}$$

(5) $$E^{11}=\frac{1+2\cdot \left(l^{F}/d^{F}\right)\cdot \eta_{l}\cdot V^{F}}{1-\eta_{l}\cdot V^{F}}\cdot E_{t}^{M};{\;\eta }_{l}=\frac{\left(E_{t}^{F}/E_{t}^{M}\right)-1}{\left(\frac{E_{t}^{F}}{E_{t}^{M}}\right)+2\cdot \left(\frac{l^{F}}{d^{F}}\right)}\;$$

(6) $$E^{22}=\frac{1+2\cdot \eta_{t}\cdot V^{F}}{1-\eta_{t}\cdot V^{F}}\cdot E_{t}^{M}\;;{\;\eta }_{t}=\frac{\left(E_{t}^{F}/E_{t}^{M}\right)-1}{\left(\frac{E_{t}^{F}}{E_{t}^{M}}\right)+2}\;$$

For the application of Halpin-Tsai equations, the mean fiber length and diameter of the fibers are required. As known, compounding and injection molding of the composites may be accompanied by a significant reduction in the fiber length and such effects may vary with the fiber content [55,56]. Such morphological changes were studied, and the results are reported in Table 2. The intrinsic Young’s modulus calculated from the Hirsch model and TP & HT model is also incorporated in Table 2.

**Figure 6:**
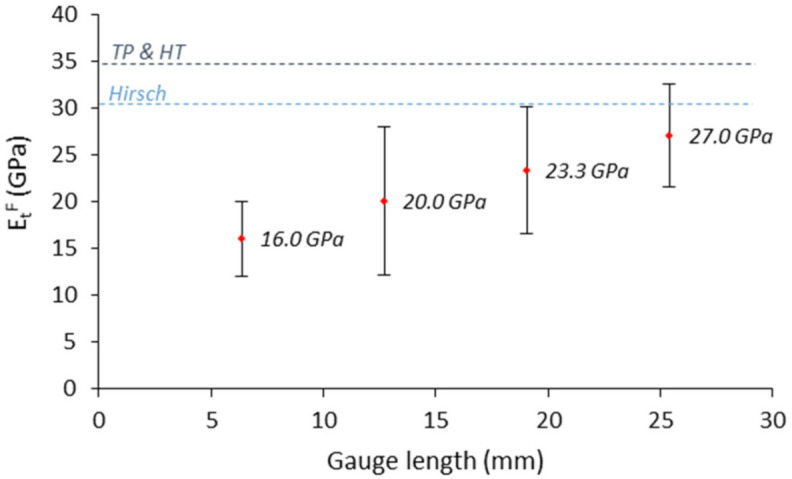
Intrinsic Young’s modulus of henequen fibers at different gauge lengths.

As hypothesized, Table 2 indicates that henequen fibers, initially chopped to 1 mm in length, were significantly shorter because of the mixing and injection molding processes. This is due to the high shear forces applied to fibers during compounding processes that lead to fiber attrition. At higher reinforcement contents, such deterioration is more pronounced because of the increment of the mixture viscosity and shearing forces. Besides, the diameter of the fibers remained very stable around 25.3–25.6 µm. It should be noted that the diameter of the initial strand was around 220 µm, which indicates that during the compounding process the single fibers were detached from the original bundle as a consequence of shearing. Overall, the average Young’s moduli of henequen fibers were set of 30.5 and 34.6 according to Hirsch and TP & HT models, respectively. In Figure 6, the estimated Young’s moduli are compared with the values obtained from the single filament tensile tests.

Results showed that with an increase in the gauge length, Young’s modulus increased from 16.0 GPa to 27.0 GPa (from 6.35 to 25.4 mm, respectively). Figure 6 also shows relatively high standard deviations, reflecting the variability in Young’s modulus measurements. Being Young’s modulus a fundamental property of the material, such differences were not expected but can be explained by the test conditions and the equipment. The measurements of the fiber deformations were made without an extensometer, and the displacement of the clamps was used to compute Young’s modulus. When the load is applied there is a slippage between the fibers and the clamps adding an error to the fiber deformation value. Such error mainly depends on the type of grips and relative slippage of the filament. The relative slippage of the filaments is expected to be more pronounced at lower gauge lengths, and thus, it could also explain the increment of Young’s modulus with the gauge length [57]. Experimental values can be corrected by isolating the fiber slippage from Young’s modulus evaluation following a method that contemplates the interactions between the testing equipment and the specimen under test. Such a method was first introduced by Guimarães et al. (1978) [58] and further reviewed by other authors [59]. The method has been typically applied for the correction of Young’s modulus of glass and carbon fibers, though, in this work the method is further considered for henequen strands. The corrected Young’s modulus ($E_{t}^{F^{*}}$) is given by Equation (7).

(7) $${\left({\mathrm{tg}\theta }_{o}\right)}^{-1}=\frac{1}{K_{m}}+\frac{1}{E_{t}^{F^{*}}}\cdot (\frac{L_{o}}{A_{o}})$$

where $\theta_{o}$ is the slope of the line resulting from the graphical representation of the force (N) and extension (mm). $K_{m}$ is a constant that represents the error generated by the testing machine. The gauge distance and cross-sectional area of the filament under test are represented by $L_{o}$ and $A_{o}$. If ${\left({\mathrm{tg}\theta }_{o}\right)}^{-1}$ is represented against $L_{o}/A_{o}$ at the different gauge lengths, it is possible to obtain the value of $K_{m}$ at the y-intercept and $E_{t}^{F^{*}}$ at the slope of the line. Therefore, the corrected Young’s modulus for henequen strands was found to be 33.3 GPa. Remarkably, this value is situated between Hirsch and TP & HT models, which suggests the good agreement between micromechanics models and the tensile testing of the strands. This contrasts with other studies which stated that micromechanics analysis and single fiber tests do not typically yield similar fiber properties [37].

At this point, the utility of micromechanics models to predict the intrinsic properties of natural fibers is underlined. Within micromechanics models, the Hirsch model does not require any input of morphological data as in the case of the Tsai-Pagano model, since it uses only experimental data from the tensile test. Overall, the Hirsch model stands as a simple, yet effective, way to estimate the intrinsic properties of natural fibers.

As expected, the intrinsic modulus is notably higher for glass fibers (18.8 GPa), though, their specific properties are in the same order because of the notably higher density of glass fibers. This makes natural fibers, and thus henequen fibers, especially attractive for low-weight material applications as for the automotive industry.

### 3.5. Modulus Efficiency, Length, and Orientations Factors

Knowing the intrinsic Young’s modulus of the fibers, it is possible to calculate the modulus efficiency factor ($\eta_{e}$) from the mRoM. The implication of the fiber length on the efficiency factor can be estimated by a modulus length factor ($\eta_{l}$) according to Cox [60] and Krenchel [61] model, as shown in Equation (8).

(8) $$\eta_{l}=1-\frac{\tan h\left(\xi \cdot l^{F}/2\right)}{\xi \cdot l^{F}/2}\;\mathrm{with},\;\xi =\frac{1}{\left(d^{F}/2\right)}\cdot \sqrt{\frac{E_{f}^{M}}{E_{f}^{F}\cdot \left(1-\upsilon \right)\cdot \sqrt{\ln\left(\pi /4V^{F}\right)}}}$$

In Equation (7), the mean fiber length and mean fiber diameter is represented by $l^{F}$ and $d^{F}$, respectively. ν stands for the Poisson’s ratio of PP (0.36). Once the modulus efficiency and length factors are computed, a modulus orientation factor ($\eta_{o}$) can be simply isolated from the following relationship: $\eta_{e}=\eta_{o}\cdot \eta_{l}$. Table 3 collects the modulus efficiency, length, and orientation factors at the different reinforcement contents.

It is observed in Table 3 that the modulus efficiency factor acquires values between 0.46 and 0.47. The values are inside the expected range for natural fiber-reinforced polymer composites, between 0.4 and 0.6 [62]. It is suggested that the fibers could effectively stiffen the composite material. The relatively high values obtained for the modulus length factor reveal the importance and significance of morphology, precisely aspect ratio, on providing stiffness to the material, being the modulus orientation factor somewhat lower, around 0.5.

## 4. Conclusions

In this work, henequen strands were chopped to 1 mm length and melt-extruded with polypropylene for the development of composite materials. A 4 wt.% of maleic anhydride grafted polypropylene was incorporated into the composites to address the issue of interfacial adhesion. Stiffness, hereby represented by Young’s modulus property, is considered a crucial parameter to glimpse the competitiveness and performance of composite materials, especially for structural applications. The results show that the addition of henequen fibers to polypropylene significantly incremented Young’s modulus from 1.5 to 6.4 GPa up to a 50 wt.% of reinforcement, corresponding to an increment of 327%. The behavior of the composites was modeled through micromechanics analysis. Micromechanics modeling provides interesting fiber properties such as intrinsic Young’s modulus, stiffening efficiency, the influence of morphology and orientation. The Young’s moduli of henequen fibers were evaluated using the Hirsch model and Tsai-Pagano model combined with Halpin-Tsai equations. The models delivered an average Young’s modulus of 30.5 and 34.6 GPa, respectively, which were found in the same order of magnitude as other natural strands such as abaca and hemp. Such values were compared to the measurements performed to the initial henequen strands through direct filament tensile testing where, after applying a correction method, a similar Young’s modulus was attained at 33.3 GPa.

## Figures and Tables

**Table 1:** Volume fraction of fibers into the composite (V^F^), Young’s modulus ($E_{t}^{C}$), and elongation at maximum stress ($\varepsilon_{t}^{C}$) of PP-based composites reinforced with 10–50 wt.% of henequen fibers containing a 4 wt.% of MAPP.

Henequen (wt.%)	V^F^	$E_{t}^{C}$ (GPa)	$\varepsilon_{t}^{C}$ (%)
0	0	1.5 ± 0.1	9.3 ± 0.2
10	0.064	2.3 ± 0.1	4.2 ± 0.1
20	0.133	3.1 ± 0.1	3.7 ± 0.1
30	0.208	4.2 ± 0.2	3.4 ± 0.2
40	0.290	5.3 ± 0.1	3.3 ± 0.2
50	0.379	6.4 ± 0.1	3.1 ± 0.1

**Table 2:** Evaluation of the mean fiber length (l_w_^F^), mean fiber diameter (d^F^), and intrinsic Young’s modulus estimated using Hirsch and TP & HT models.

Henequen (wt.%)	V^F^	l_w_^F^ (µm)	d^F^ (µm)	$E_{t}^{F}—\mathrm{Hirsch}$ (GPa)	$E_{t}^{F}—\mathrm{TP}\&\mathrm{HT}$ (GPa)
10	0.064	826.3 ± 10.6	25.5 ± 0.1	30.5	36.5
20	0.133	784.2 ± 12.3	25.6 ± 0.1	29.1	33.4
30	0.208	741.9 ± 5.9	25.3 ± 0.2	31.3	36.1
40	0.290	708.1 ± 17.2	25.4 ± 0.2	31.3	34.8
50	0.379	674.3 ± 7.2	25.5 ± 0.1	30.5	32.0
			Mean value	30.5 ± 0.9	34.6 ± 1.9

l_w_^F^: Weight-weighted means fiber length.

**Table 3:** Modulus efficiency (η_e_), length (η_l_), and orientation (η_o_) factors for the henequen-reinforced PP composites.

Henequen (wt.%)	η_e_	η_l_	η_o_
10	0.46	0.88	0.52
20	0.46	0.89	0.52
30	0.46	0.90	0.52
40	0.47	0.91	0.51
50	0.47	0.92	0.52

## Data Availability

The data presented in this study are available on request from the corresponding author.

## References

[1] Thyavihalli Girijappa Y.G., Mavinkere Rangappa S., Parameswaranpillai J., Siengchin S. (2019). Natural Fibers as Sustainable and Renewable Resource for Development of Eco-Friendly Composites: A Comprehensive Review. Front. Mater..

[2] Sanjay M.R., Siengchin S., Parameswaranpillai J., Jawaid M., Pruncu C.I., Khan A. (2019). A comprehensive review of techniques for natural fibers as reinforcement in composites: Preparation, processing and characterization. Carbohydr. Polym..

[3] Fitzgerald A., Proud W., Kandemir A., Murphy R.J., Jesson D.A., Trask R.S., Hamerton I., Longana M.L. (2021). A life cycle engineering perspective on biocomposites as a solution for a sustainable recovery. Sustainability.

[4] Ferrero B., Fombuena V., Fenollar O., Boronat T., Balart R. (2008). Development of Natural Fiber-Reinforced Plastics (NFRP) Based on Biobased Polyethylene and Waste Fibers From Posidonia oceanica Seaweed. Polym. Polym. Compos..

[5] Wu Y., Xia C., Cai L., Garcia A.C., Shi S.Q. (2018). Development of natural fiber-reinforced composite with comparable mechanical properties and reduced energy consumption and environmental impacts for replacing automotive glass-fiber sheet molding compound. J. Clean. Prod..

[6] Xu A., Wang Y., Gao J., Wang J. (2019). Facile fabrication of a homogeneous cellulose/polylactic acid composite film with improved biocompatibility, biodegradability and mechanical properties. Green Chem..

[7] Serra-Parareda F., Vilaseca F., Espinach F.X., Mutjé P., Delgado-Aguilar M., Tarrés Q. (2021). Stiffening potential of lignocellulosic fibers in fully biobased composites: The case of abaca strands, spruce tmp fibers, recycled fibers from onp and barley tmp fibers. Polymers.

[8] Peças P., Carvalho H., Salman H., Leite M. (2018). Natural Fibre Composites and Their Applications: A Review. J. Compos. Sci..

[9] Oliver-Ortega H., Julian F., Espinach F.X., Tarrés Q., Ardanuy M., Mutjé P. (2019). Research on the use of lignocellulosic fibers reinforced bio-polyamide 11 with composites for automotive parts: Car door handle case study. J. Clean. Prod..

[10] Karmaker A.C., Hoffmann A., Hinrichsen G. (1994). Influence of water uptake on the mechanical properties of jute fiber-reinforced polypropylene. J. Appl. Polym. Sci..

[11] Carus M., Eder A., Dammer L., Korte H., Scholz L., Essel R., Breitmayer E., Barth M. (2015). WPC/NFC Market Study 2014-10 (Update 2015-06): Wood-Plastic Composites (WPC) and Natural Fibre Composites (NFC): European and Global Markets 2012 and Future Trends in Automotive and Construction. Bio-Based Eu/Mark..

[12] Bledzki A.K., Gassan J., Theis S. (1998). Wood-filled thermoplastic composites. Mech. Compos. Mater..

[13] Gironès J., Lopez J.P., Vilaseca F., Bayer R., Herrera-Franco P.J., Mutjé P. (2011). Biocomposites from Musa textilis and polypropylene: Evaluation of flexural properties and impact strength. Compos. Sci. Technol..

[14] Baley C. (2002). Analysis of the flax fibres tensile behaviour and analysis of the tensile stiffness increase. Compos. Part A Appl. Sci. Manuf..

[15] Serra A., Tarrés Q., Llop M., Reixach R., Mutjé P., Espinach F.X. (2019). Recycling dyed cotton textile byproduct fibers as polypropylene reinforcement. Text. Res. J..

[16] Hyvärinen M., Kärki T. (2015). The Effects of the Substitution of Wood Fiberwith Agro-based Fiber (Barley Straw) on the Properties of Natural Fiber/Polypropylene Composites. MATEC Web of Conferences.

[17] Reixach R., Espinach F.X., Arbat G., Julián F., Delgado-Aguilar M., Puig J., Mutjé P. (2015). Tensile properties of polypropylene composites reinforced with mechanical, thermomechanical, and chemi-thermomechanical pulps from orange pruning. BioResources.

[18] Delgado-Aguilar M., González I., Pèlach M.A., De La Fuente E., Negro C., Mutjé P. (2015). Improvement of deinked old newspaper/old magazine pulp suspensions by means of nanofibrillated cellulose addition. Cellulose.

[19] Sanadi A.R., Young R.A., Clemons C., Rowell R.M. (1994). Recycled Newspaper Fibers as Reinforcing Fillers in Thermoplastics: Part I-Analysis of Tensile and Impact Properties in Polypropylene. J. Reinf. Plast. Compos..

[20] Gholampour A., Ozbakkaloglu T. (2020). A Review of Natural Fiber Composites: Properties, Modification and Processing Techniques, Characterization, Applications.

[21] Fajardo Cabrera de Lima L.D.P., Chamorro Rodríguez C.D., Mina Hernandez J.H. (2021). Use of Organic Acids in Bamboo Fiber-Reinforced Polypropylene Composites: Mechanical Properties and Interfacial Morphology. Polymers.

[22] Faruk O., Bledzki A.K., Fink H.-P., Sain M. (2012). Biocomposites reinforced with natural fibers: 2000–2010. Prog. Polym. Sci..

[23] Xie Y., Hill C.A.S., Xiao Z., Militz H., Mai C. (2010). Silane coupling agents used for natural fiber/polymer composites: A review. Compos. Part A Appl. Sci. Manuf..

[24] Cruz J., Fangueiro R. (2016). Surface modification of natural fibers: A review. Procedia Eng..

[25] Keener T.J., Stuart R.K., Brown T.K. (2004). Maleated coupling agents for natural fibre composites. Compos. Part A Appl. Sci. Manuf..

[26] Zabihzadeh S.M., Ebrahimi G., Enayati A.A. (2011). Effect of Compatibilizer on Mechanical, Morphological, and Thermal Properties of Chemimechanical Pulp-reinforced PP Composites. J. Thermoplast. Compos. Mater..

[27] Pimenta M.T.B., Carvalho A.J.F., Vilaseca F., Girones J., López J.P., Mutjé P., Curvelo A.A.S. (2008). Soda-treated sisal/polypropylene composites. J. Polym. Environ..

[28] Sombatsompop N., Yotinwattanakumtorn C., Thongpin C. (2005). Influence of type and concentration of maleic anhydride grafted polypropylene and impact modifiers on mechanical properties of PP/wood sawdust composites. J. Appl. Polym. Sci..

[29] Silva N.G., Cortat L.I., Mulinari D.R. (2021). Effect of Alkaline Treatment and Coupling Agent on Thermal and Mechanical Properties of Macadamia Nutshell Residues Based PP Composites. J. Polym. Environ..

[30] Soleimani M., Tabil L., Panigrahi S., Opoku A. (2008). The effect of fiber pretreatment and compatibilizer on mechanical and physical properties of flax fiber-polypropylene composites. J. Polym. Environ..

[31] Nayak S.K., Dixit G., Appukuttan K.K. (2012). Sisal fiber (SF) reinforced recycled polypropylene (RPP) composites. Int. J. Plast. Technol..

[32] Neagu R.C., Gamstedt E.K., Berthold F. (2006). Stiffness contribution of various wood fibers to composite materials. J. Compos. Mater..

[33] Oliver-Ortega H., Granda L.A., Espinach F.X., Delgado-Aguilar M., Duran J., Mutjé P. (2016). Stiffness of bio-based polyamide 11 reinforced with softwood stone ground-wood fibres as an alternative to polypropylene-glass fibre composites. Eur. Polym. J..

[34] Facca A.G., Kortschot M.T., Yan N. (2006). Predicting the elastic modulus of natural fibre reinforced thermoplastics. Compos. Part A Appl. Sci. Manuf..

[35] Rodriguez M., Rodriguez A., Bayer J., Vilaseca F., Girones J., Mutje P. (2010). Determination of corn stalk fibers’ strength through modeling of the mechanical properties of its composites. BioResources.

[36] Schmachtenberg E., Brandt M. (2006). Mechanical design of injection moulded parts made of short-fibre reinforced thermoplastics by means of integrative simulation. J. Polym. Eng..

[37] Shah D.U., Nag R.K., Clifford M.J. (2016). Why do we observe significant differences between measured and “back-calculated” properties of natural fibres?. Cellulose.

[38] Tarrés Q., Vilaseca F., Herrera-Franco P.J., Espinach F.X., Delgado-Aguilar M., Mutjé P. (2019). Interface and micromechanical characterization of tensile strength of bio-based composites from polypropylene and henequen strands. Ind. Crops Prod..

[39] Chihaoui B., Serra-Parareda F., Tarrés Q., Espinach F.X., Boufi S., Delgado-Aguilar M. (2020). Effect of the fiber treatment on the stiffness of date palm fiber reinforced PP composites: Macro and micromechanical evaluation of the young’s modulus. Polymers.

[40] Mohanty S., Nayak S.K., Verma S.K., Tripathy S.S. (2004). Effect of MAPP as a Coupling Agent on the Performance of Jute-PP Composites. J. Reinf. Plast. Compos..

[41] Siregar J.P., Jaafar J., Cionita T., Jie C.C., Bachtiar D., Rejab M.R.M., Asmara Y.P. (2019). The Effect of Maleic Anhydride Polyethylene on Mechanical Properties of Pineapple Leaf Fibre Reinforced Polylactic Acid Composites. Int. J. Precis. Eng. Manuf. Green Technol..

[42] Nayak S.K., Mohanty S. (2010). Sisal Glass Fiber Reinforced PP Hybrid Composites: Effect of MAPP on the Dynamic Mechanical and Thermal Properties. J. Reinf. Plast. Compos..

[43] Brahmakumar M., Pavithran C., Pillai R.M. (2005). Coconut fibre reinforced polyethylene composites: Effect of natural waxy surface layer of the fibre on fibre/matrix interfacial bonding and strength of composites. Compos. Sci. Technol..

[44] Prasad S.V., Pavithran C., Rohatgi P.K. (1983). Alkali treatment of coir fibres for coir-polyester composites. J. Mater. Sci..

[45] Arrakhiz F.Z., El Achaby M., Malha M., Bensalah M.O., Fassi-Fehri O., Bouhfid R., Benmoussa K., Qaiss A. (2013). Mechanical and thermal properties of natural fibers reinforced polymer composites: Doum/low density polyethylene. Mater. Des..

[46] Serrano A., Espinach F.X., Tresserras J., Pellicer N., Alcala M., Mutje P. (2014). Study on the technical feasibility of replacing glass fibers by old newspaper recycled fibers as polypropylene reinforcement. J. Clean. Prod..

[47] Thomason J.L., Rudeiros-Fernández J.L. (2018). A review of the impact performance of natural fiber thermoplastic composites. Front. Mater..

[48] Kandemir A., Longana M.L., Panzera T.H., del Pino G.G., Hamerton I., Eichhorn S.J. (2021). Natural fibres as a sustainable reinforcement constituent in aligned discontinuous polymer composites produced by the HiPerDiF method. Materials.

[49] López J.P., Mutjé P., Pèlach M.À., Mansouri N.-E., Boufi S., Vilaseca F. (2012). Analysis of the tensile modulus of polypropylene composites reinforced with stone groundwood fibers. BioResources.

[50] Hirsch T.J. (1962). Modulus of Elasticity of Concrete Affected by Elastic Moduli of Cement Paste Matrix and Aggregate. J. Proc..

[51] Kalaprasad G., Joseph K., Thomas S., Pavithran C. (1997). Theoretical modelling of tensile properties of short sisal fibre-reinforced low-density polyethylene composites. J. Mater. Sci..

[52] Affdl J.H., Kardos J.L. (1976). The Halpin-Tsai equations: A review. Polym. Eng. Sci..

[53] Halpin J.C. (1969). Effects of Environmental Factors on Composite Materials.

[54] Halpin J.C., Pagano N.J. (1969). The Laminate Approximation for Randomly Oriented Fibrous Composites. J. Compos. Mater..

[55] Li Y., Pickering K.L., Farrell R.L. (2009). Determination of interfacial shear strength of white rot fungi treated hemp fibre reinforced polypropylene. Compos. Sci. Technol..

[56] Bledzki A.K., Gassan J. (1999). Composites reinforced with cellulose based fibers. Prog. Polym. Sci..

[57] de Andrade Silva F., Chawla N., de Toledo Filho R.D. (2008). Tensile behavior of high performance natural (sisal) fibers. Compos. Sci. Technol..

[58] Guimarães J.R., Chawla K.K. (1978). Characteristics of the stiffness of a universal tensile testing apparatus. Metalurgia.

[59] Pardini L.C., Manhani L.G.B. (2002). Influence of the testing gage length on the strength, Young’s modulus and Weibull modulus of carbon fibres and glass fibres. Mater. Res..

[60] Cox H.L. (1952). The elasticity and strength of paper and other fibrous materials. Br. J. Appl. Phys..

[61] Krenchel H. (1964). Fibre Reinforcement. Theoretical and Practical Investigations of the Elasticity and Strength of Fibre-Reinforced Materials.

[62] Jiménez A.M., Delgado-Aguilar M., Tarrés Q., Quintana G., Fullana-i-Palmer P., Mutjè P., Espinach F.X. (2017). Sugarcane bagasse reinforced composites: Studies on the young’s modulus and macro and micro-mechanics. BioResources.

